# Prevalence and Risk Factors of Fluoroquinolone Resistance in Major Bacterial Pathogens: A Systematic Review and Meta‐Analysis

**DOI:** 10.1155/ijm/3173058

**Published:** 2026-04-28

**Authors:** I Made Bayu Anggriawan, Ike Dhiah Rochmawati

**Affiliations:** ^1^ School of Medicine, Dentistry and Nursing, University of Glasgow, Glasgow, UK, gla.ac.uk; ^2^ Rumah Sakit Umum Pusat Prof. I.G.N.G. Ngoerah, Denpasar, Indonesia; ^3^ Lembaga Pengelola Dana Pendidikan, Central Jakarta, Jakarta, Indonesia; ^4^ School of Health and Wellbeing, University of Glasgow, Glasgow, UK, gla.ac.uk; ^5^ Faculty of Pharmacy, University of Surabaya, Surabaya, Indonesia, ubaya.ac.id

**Keywords:** antimicrobial resistance, *Escherichia coli*, fluoroquinolone resistance, *Klebsiella* spp., *Mycobacterium tuberculosis*, odds ratio: meta-analysis, prevalence, *Proteus* spp., *Pseudomonas aeruginosa*, risk factors, systematic review

## Abstract

**Background:**

The widespread use of fluoroquinolones for the treatment of Gram‐negative bacterial infections has contributed to the rising prevalence of antimicrobial resistance. However, limited studies have systematically analyzed the prevalence of fluoroquinolone resistance (FQR) and its associated risk factors.

**Methods:**

A systematic review and meta‐analysis were conducted, screening studies published between January 1, 2014, and October 31, 2024, from the PubMed, Medline, Embase, and CINAHL databases. Studies were included based on the following criteria: observational designs, evaluation of Gram‐negative bacteria for FQR in human subjects, and investigation of FQR and associated risk factors. Data analysis, including pooled prevalence estimation and odds ratio calculation, was performed using R Studio (Version 4.2.3) with the metafor package. Heterogeneity among studies was assessed using *Q* and *I* ^2^ statistics. A funnel plot was used to assess potential publication bias among the included studies.

**Results:**

A total of 24 studies were included in the systematic review and meta‐analysis. The pooled prevalence of FQR across pathogens was 35% (95% CI: 30%–40%), with species‐specific rates of *Campylobacter* spp. (49%), *Escherichia coli* (35%), *Klebsiella* spp. (23%), *Mycobacterium tuberculosis* (40%), *Pseudomonas aeruginosa* (34%), *Proteus* spp. (45%), and others (26%). Subgroup analyses showed variation by fluoroquinolone generation and year of publication, with *E. coli* displaying increasing resistance trends over time. Risk factors significantly associated with FQR included the presence of an indwelling catheter, advanced age, prior hospitalization, previous fluoroquinolone or other antibiotic use, drug‐resistant TB, international travel, sex, and unfavorable treatment outcomes.

**Discussion:**

Although the overall prevalence of FQR was modest, increasing trends in *E. coli* and the presence of multiple associated risk factors highlight important clinical implications. These findings emphasize the need to consider prior antibiotic use and hospitalization when guiding treatment and antimicrobial stewardship. However, these results should be interpreted with caution due to high heterogeneity and variability across the included studies.

## 1. Introduction

The prevalence of antibacterial resistance is increasing worldwide. Previous studies found a high rate of several kinds of antibiotic resistance in different organisms. Fluoroquinolones are well‐known antibiotics for treating some infections, such as pulmonary, sexually transmitted, and gastrointestinal infections. Antimicrobial resistance (AMR) of fluoroquinolones is found mostly in Gram‐negative bacteria (GNBs), where the mechanism involves mutations in the target enzymes, DNA gyrase, and topoisomerase IV, as well as modifications in efflux pumps, porins, and plasmid‐mediated quinolone resistance [1].

Plasmid‐mediated transfer of resistance and nosocomial transmission of GNBs, particularly Enterobacteriaceae, *Pseudomonas aeruginosa*, and *Acinetobacter baumannii*, have led to widespread dissemination, outbreaks, and untreatable infections [2, 3]. A retrospective cohort study (2018) at 173 hospitals in the United States aimed to determine the prevalence of difficult‐to‐treat resistance (DTR) among GNB infections. This impacted the mortality rate among patients with GNB infections, which was 20% higher with nonresistant GNBs [4].

*Klebsiella pneumoniae*, another opportunistic pathogen, primarily affects the urinary tract, respiratory system, and bloodstream, particularly in immunocompromised individuals. It has developed resistance to multiple antibiotic classes [5]. On the other hand, pathogenic strains of *Escherichia coli* are known to cause food poisoning and urinary tract infections (UTIs). The WHO Global Antimicrobial Resistance and Use Surveillance System (GLASS) report 2022 highlights that both ciprofloxacin and levofloxacin face serious resistance challenges in *E. coli*, with global medians around 35%–45% [6]. These bacteria have exhibited widespread resistance across most antibiotic classes, particularly fluoroquinolones [7].

Furthermore, fluroquinolones have been effectively used as a second line of tuberculosis (TB) treatment since 1984. Various studies of FQR are reported to be associated with poor medication treatment in TB [8], especially for the patient with multidrug‐resistant (MDR) TB. Although *Mycobacterium tuberculosis* is taxonomically distinct from GNBs, it was included due to the central role of fluoroquinolones across multiple bacterial infections [9]. A study in Pakistan (2016) showed that the prevalence of ofloxacin resistance was relatively higher in TB treatment, accounting for 52.7% [10]. Importantly, the World Health Organization identifies fluoroquinolones as essential components of MDR treatment [11], emphasizing their critical clinical importance. Resistance to fluoroquinolones in *M. tuberculosis* is a growing global concern and is associated with poorer treatment outcomes.

The gradual increase of fluoroquinolones makes it an alarming issue for global health. The wide intercountry variability indicates that some countries still report very high resistance levels, while several others lack prevalence estimates of FQR, particularly in GNBs. Moreover, the pooled estimates of FQR in GNBs and other major pathogens are presently unknown. Therefore, this systematic review and meta‐analysis are aimed at providing an update on the rise of FQR in GNBs.

## 2. Materials and Methods

The protocol of this systematic review and meta‐analysis was registered on the International Prospective Register of Systematic Reviews (PROSPERO) with ID: CRD420251023485 and reported based on the Preferred Reporting Items for Systematic Review and Meta‐Analysis statement (PRISMA) guideline.

### 2.1. Data Source and Searching Strategy

Data about FQR in GNBs were gathered using electronic databases and search engines. A comprehensive systematic search was conducted between November 2, 2024, and November 27, 2024, across the PubMed, Medline, Embase, and CINAHL databases, covering all available publications from January 1, 2014, to October 31, 2024. The complete search strategy for all databases is explained in Table S1.

### 2.2. Study Selection and Quality Assessment

All the studies that were retrieved were exported into the Rayyan.Ai to manage reference and study duplication. Reviewers (IMB and IDR) independently screened the titles, abstracts, and full texts to determine the eligibility of each study. Where there was disagreement, a decision was held to resolve any discrepancy by achieving consensus and to include articles in the final analysis. The Newcastle–Ottawa Scale (NOS) was used to assess the risk of bias of observational studies (Table S1).

### 2.3. Eligibility Criteria

All the retrieved papers from the databases were screened for eligibility. Papers included in the analysis should follow these criteria: (i) studies involving patients with GNB infection and the rate of FQR. Studies on *M. tuberculosis* were also included despite taxonomic differences due to the shared mechanism of action of fluoroquinolones and the clinical importance of fluoroquinolone resistance in MDR TB management; (ii) the primary outcome of interest was the prevalence of FQR, while the secondary outcome was the factors associated with FQR; (iii) observational studies; (iv) studies conducted in hospital settings (e.g., those that recorded medications prescribed to discharged patients), primary care settings, or multicenter registries; and (v) studies published in the English language.

Papers were excluded if they reported (i) antibiotic resistance other than fluroquinolones; (ii) studies involving organisms other than GNBs; and (iii) studies classified as reviews (literature reviews, narrative reviews, and systematic reviews), meta‐analyses, letters, correspondences, or conference abstracts.

Antimicrobial susceptibility testing methods and resistance breakpoints varied across studies. Most studies reported using internationally recognized standards such as Clinical and Laboratory Standards Institute (CLSI) and the European Committee on Antimicrobial Susceptibility Testing (EUCAST) guidelines. Because breakpoint criteria have evolved over time and differ slightly between these systems, some variability in resistance classification may have occurred.

### 2.4. Screening and Data Extraction

Two investigators reviewed the articles included and recorded relevant data independently using data extraction tools that were prepared in Microsoft Excel. Disagreements were resolved through discussion and consensus between the reviewers. When necessary, discrepancies were re‐evaluated by revisiting the original articles to ensure accurate inclusion and data extraction. PRISMA diagram was followed for study selection. Data extraction was done and tabulated into the general characteristics of the included studies. The following data were extracted from each original article: author′s name, year of publication, study country, study design, sample size, number of bacteria isolates tested for FQR, and the prevalence of FQR.

### 2.5. Outcome Measurement

The FQR was defined as resistance to any generation, such as first generation (nalidixic acid), second generation (ciprofloxacin, norfloxacin), third generation (levofloxacin), and fourth generation (moxifloxacin).

### 2.6. Data Analysis

The data that had already been extracted were analyzed using R Studio Version 4.2.4 with the metafor package [12, 13]. The overall prevalence of FQR was tested among pathogens using a random‐effects model, due to the heterogeneity of data. The statistical test was also conducted for the heterogeneity by the examination of *Q* and *I* ^2^. An *I* ^2^ value of 0% indicated true homogeneity, while values of 25%, 50%, and 75% represented low, moderate, and high heterogeneity, respectively [14]. A *p* value of less than 0.05 was used to declare the presence of heterogeneity. Subgroup analyses were performed to evaluate the prevalence and temporal trends of FQR in *E. coli* across second‐ and third‐generation fluoroquinolones, as well as by year of publication.

Risk factors associated with FQR were also analyzed using this package. Where available, odds ratios (ORs) with 95% confidence intervals (CIs) were extracted or calculated, and meta‐analyses were conducted under a random‐effects model to identify potential determinants of resistance. Publication bias was checked by the funnel plot analysis [15].

## 3. Results

### 3.1. Literature Search and Selection Process

**Figure 1:**
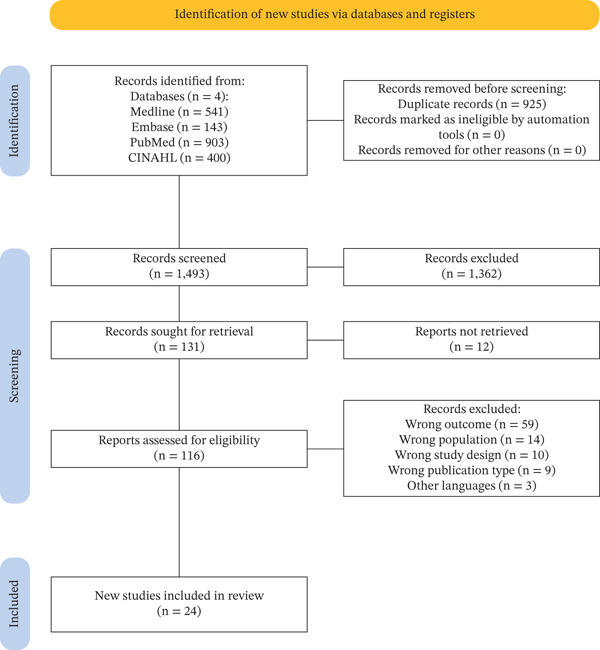
PRISMA flowchart for the selection study process.

A total of 1987 articles were retrieved from selected databases. After removal of duplicates, 1493 articles remained for the screening process. Title and abstract screening yielded 125 articles for full‐text assessment. A total of 24 studies were included in this systematic review and meta‐analysis. The process of study selection can be shown in the PRISMA flowchart, as seen in Figure 1.

### 3.2. Characteristic of Included Studies

A total of 24 studies were included and quantitatively analyzed (Table S2). Most included studies employed a retrospective design, collecting data primarily from hospital‐based settings, although several were conducted in an outpatient setting. Diagnosis of resistance relied on culture and sensitivity testing of bacterial isolates, as described in the methodology of each included study.

**Figure 2:**
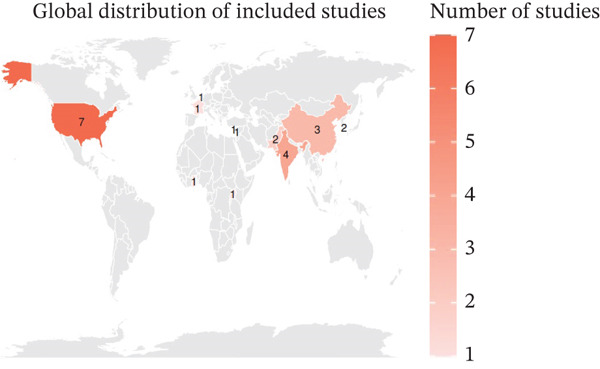
Global distribution of studies included in the meta‐analysis. The orange color corresponds to the number of studies contributed by each country. The majority of studies originated from the United States and Asia, with limited representation from Africa and Europe.

The included studies demonstrated a broad geographical distribution (Figure 2), with the highest representation from the United States, followed by India and China. Additional studies were contributed by countries across Europe, Asia, and Africa, including Pakistan, South Korea, France, Ghana, Uganda, Lebanon, Cyprus, and the Netherlands. This distribution reflects a diverse range of healthcare settings and antimicrobial use patterns, although the overrepresentation of certain regions may contribute to heterogeneity in the pooled estimates.

The age of participants, when reported, generally covered a broad range but frequently centered on adults in middle to older age groups. Studies originated from a wide geographic distribution, reflecting a global concern regarding AMR. The study settings varied from large national surveillance datasets to smaller (details shown in Table 1), single‐center cohorts, highlighting both population‐level and institution‐specific perspectives. The characteristics of the included studies are shown in Table S2.

**Table 1:** The variety of surveillance data from the included studies.

No.	Authors	Year of publication	Country	Number of patients
1	Brintz et al. [16]	2024	United States	214,656
2	Martinet et al. [17]	2024	France	210
3	Marepalli et al. [18]	2024	India	280
4	Li et al. [19]	2024	China	850
5	Baek et al. [20]	2024	South Korea	108
6	Chhakchhuak et al. [21]	2023	India	586
7	Braam et al. [22]	2022	Netherland	669
8	Faine et al. [23]	2022	United States	3779
9	Deku et al. [24]	2022	Ghana	135
10	John et al. [25]	2021	United States	8680
11	Rodrigues et al. [26]	2021	United States	214
12	Odoki et al. [27]	2020	Uganda	86
13	Zilberberg et al. [28]	2020	United States	23,331
14	Jouhar et al. [29]	2020	Lebanon	356
15	Ruh et al. [30]	2019	Cyprus	500
16	Kim et al. [31]	2019	South Korea	130
17	Poonia et al. [32]	2018	India	100
18	Zaidi et al. [33]	2017	Pakistan	133
19	Banukumar and Sukumar [34]	2017	India	718
20	Talan et al. [35]	2016	United States	521
21	Bidell et al. [36]	2016	United States	9944
22	Tian et al. [37]	2016	China	508
23	Ahmad et al. [10]	2015	Pakistan	243
24	Zhang et al. [38]	2015	China	38

**Figure 3:**
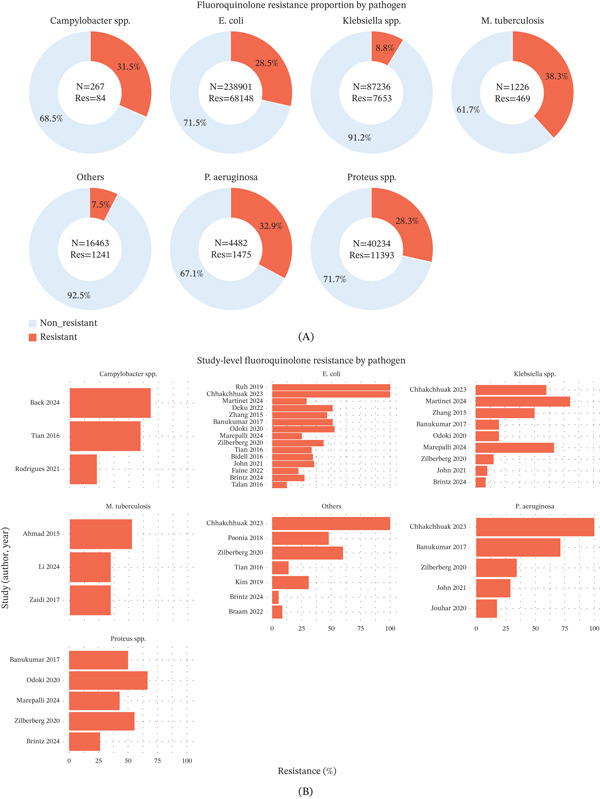
Proportion of FQR across major bacterial pathogens from included studies. (A) Donut charts illustrate the proportion of resistant and nonresistant isolates for each pathogen included in the meta‐analysis. Each chart represents 100% of the total isolates for the respective organism. The orange segment indicates FQR isolates, while the grey segment represents nonresistant isolates. The total number of isolates (*N*) and the number of resistant isolates (Res) are displayed at the center of each chart. Percentage values indicate the proportion of resistant and nonresistant isolates within each pathogen group. (B) Bar charts show the percentage of FQR in each included study. Details of the value from each study are available in Table S3.

A notable pattern of AMR in several bacterial pathogens is shown in Figure 3 and Table S3. The resistance testing for fluoroquinolones in this meta‐analysis included a total of 388,809 pathogen isolates, consisting of 238,901 *E. coli*; 87,236 *Klebsiella* spp.; 267 *Campylobacter* spp.; 1226 *M. tuberculosis*; 4482 *P. aeruginosa*; 40,234 *Proteus* spp.; and 16,463 isolates grouped as “other” (e.g., *Salmonella typhi*, *Stenotrophomonas maltophilia*, *Providencia* spp., *Mycoplasma genitalium*, *Acinetobacter baumannii*, and *Citrobacter* spp.). Across three studies, resistance in *Campylobacter* spp. ranged from approximately 23% to 68%, with the highest levels reported from South Korea. Overall, 267 isolates were tested, with a resistance prevalence of 31.5%.

*Escherichia coli* resistance was evaluated in 14 studies, comprising over 230,000 isolates. Resistance rates varied widely by region and sample size, from low to high prevalence (up to 100%). Particularly, high resistance rates were observed in studies from India and Cyprus. A large‐scale study in the United States contributed the majority of data, reporting resistance in approximately 27% of over 213,000 isolates. Four studies assessed *Klebsiella* spp., with resistance observed in 15.4% of the 3467 total isolates. Most data originated from a US‐based surveillance study, while smaller studies in Asia and Africa reported variable but generally modest resistance levels.

### 3.3. Meta‐Analysis

**Figure 4:**
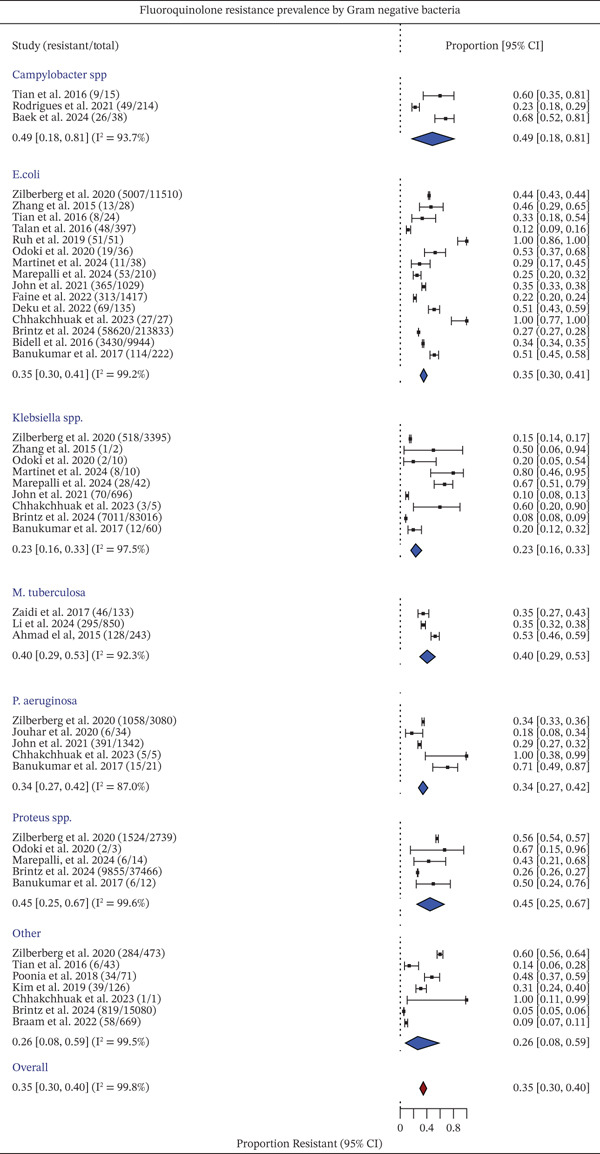
Pooled prevalence of FQR in major bacterial pathogens. Forest plots present the proportion of FQR isolates across individual studies and bacterial species. Each square represents the point estimate of resistance prevalence for a study, with horizontal lines indicating the 95% confidence intervals (CIs). The size of the square reflects the relative weight of each study. Pooled estimates for each pathogen are shown as blue diamonds, while the overall pooled prevalence is represented by a red diamond. Substantial heterogeneity was observed across studies, as indicated by high *I* ^2^ values. The dashed vertical line represents the overall pooled estimate.

The pooled prevalence of FQR and heterogeneity for each GNB were assessed and are shown in Figure 4. *Campylobacter* spp. showed a pooled resistance of 0.49 (95% CI: 0.18–0.81), with high heterogeneity (*I* ^2^ = 93.7*%*). *Escherichia coli* had a pooled resistance estimate of 0.35, driven by wide‐ranging prevalence values, from 12% to 100%, and significant heterogeneity across studies (*I* ^2^ = 99.2*%*). *Klebsiella* spp. showed a pooled resistance of 0.23, with heterogeneity at *I* ^2^ = 97.5*%*. Other pathogens, such as *P. aeruginosa*, *Proteus* spp., and *M. tuberculosis*, had respective pooled resistance estimates of 0.34, 0.45, and 0.40, all with high heterogeneity (*I* ^2^ > 87*%*).

Heterogeneity was very high (*I* ^2^ = 99.8*%*), consistent with the inherent variation across bacterial species, geographic regions, clinical settings, and laboratory methods. Such high heterogeneity is typical in AMR prevalence meta‐analyses and reflects true underlying differences rather than methodological bias [7, 39].

### 3.4. Subgroup Analysis

Having significant heterogeneity of FQR to GNBs across the included studies, a subgroup analysis was conducted to evaluate the pooled prevalence and time trends for resistance to second‐ and third‐generation fluoroquinolones in *E. coli* (Table 2).

**Table 2:** Subgroup analysis of fluoroquinolone generation–specific resistance in *E. coli.*

Fluoroquinolone	Pooled prevalence		Time trend (prevalence increase overtime)			
	Prevalence	95% CI	Regression coefficient	95% CI	*p*value	Moderator test
Ciprofloxacin	46.77%	25.13%–68.41%	0.16	−0.16 to 0.49	0.32	0.99
Norfloxacin	62.98%	27.51%–98.45%	0.36	−0.86 to 1.58	0.56	0.33
Levofloxacin	38.92%	7.94%–69.90%	0.42	0.05–0.79	0.02	5.14

*E. coli* was selected for subgroup analysis due to its high representation across studies, allowing for more reliable and interpretable estimates, whereas data for other pathogens were insufficient for meaningful subgroup evaluation.

#### 3.4.1. Ciprofloxacin

**Figure 5:**
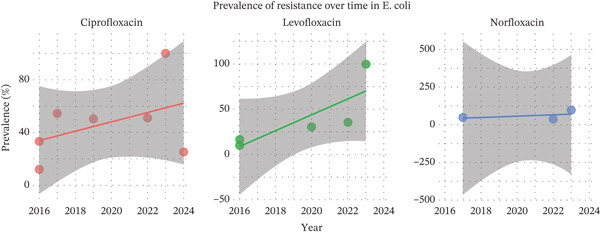
Prevalence of *E. coli* over time from ciprofloxacin, levofloxacin, and norfloxacin.

The pooled prevalence of ciprofloxacin resistance in *E. coli* was 46.8%. The regression coefficient (*β*) for the time trend was 0.16 (*p* = 0.5602; 95% CI: −0.86 to 1.58), indicating a positive but nonsignificant association between year and resistance prevalence. This suggests a slight increase in resistance over time (Figure 5); however, the change was not statistically significant.

#### 3.4.2. Norfloxacin

The pooled prevalence of *E. coli* resistance to norfloxacin was 63%. The regression coefficient (*β*) for the time trend was 0.29 (*p* = 0.1288; 95% CI: −0.0847 to 0.6673), indicating a modest positive association between year and resistance prevalence (Figure 5), though this was not statistically significant.

#### 3.4.3. Levofloxacin

The pooled prevalence of *E. coli* resistance to levofloxacin was 39%. The regression coefficient (*β*) for the time trend was 0.43 (*p* = 0.0233; 95% CI: 0.056–0.79), indicating a statistically significant positive association between year and resistance prevalence (Figure 5), suggesting an increasing trend in resistance over time.

### 3.5. Risk Factor Analysis

Several factors associated with FQR were evaluated in this review, including demographic, treatment‐related, and travel history variables. Some risk factors were evaluated based on a limited number of studies, such as catheter use and age (*n* = 2), while others included only three to four studies. This limited evidence base may have contributed to wide CIs and reduced precision of the pooled estimates. Two demographic factors were analyzed, but neither showed a statistically significant association with FQR. Age was associated with only a 4% increase in odds (OR = 1.04; 95% CI: 0.65–1.67; *p* = 0.9099), and sex also showed no significant association (OR = 1.01; 95% CI: 0.76–1.33; *p* = 0.6663).

In terms of treatment and care‐related factors, six variables were examined: indwelling catheter use, history of hospitalization, prior antibiotic use (e.g., fluoroquinolones), drug‐resistant TB, and unfavorable treatment outcomes. Among these, a history of hospitalization (OR = 2.18; 95% CI: 1.23–3.86) and prior fluoroquinolone use (OR = 3.99; 95% CI: 1.31–12.18) were found to be significantly associated with FQR. Although drug‐resistant TB (OR = 2.33; 95% CI: 0.85–6.35) and previous antibiotic use (OR = 1.48; 95% CI: 0.76–2.89) showed increased odds of FQR, these associations were not statistically significant. Similarly, the presence of an indwelling catheter (OR = 2.10; 95% CI: 0.81–5.43) and unfavorable treatment outcomes (OR = 1.44; 95% CI: 0.36–5.85) did not show a significant relationship with FQR.

**Figure 6:**
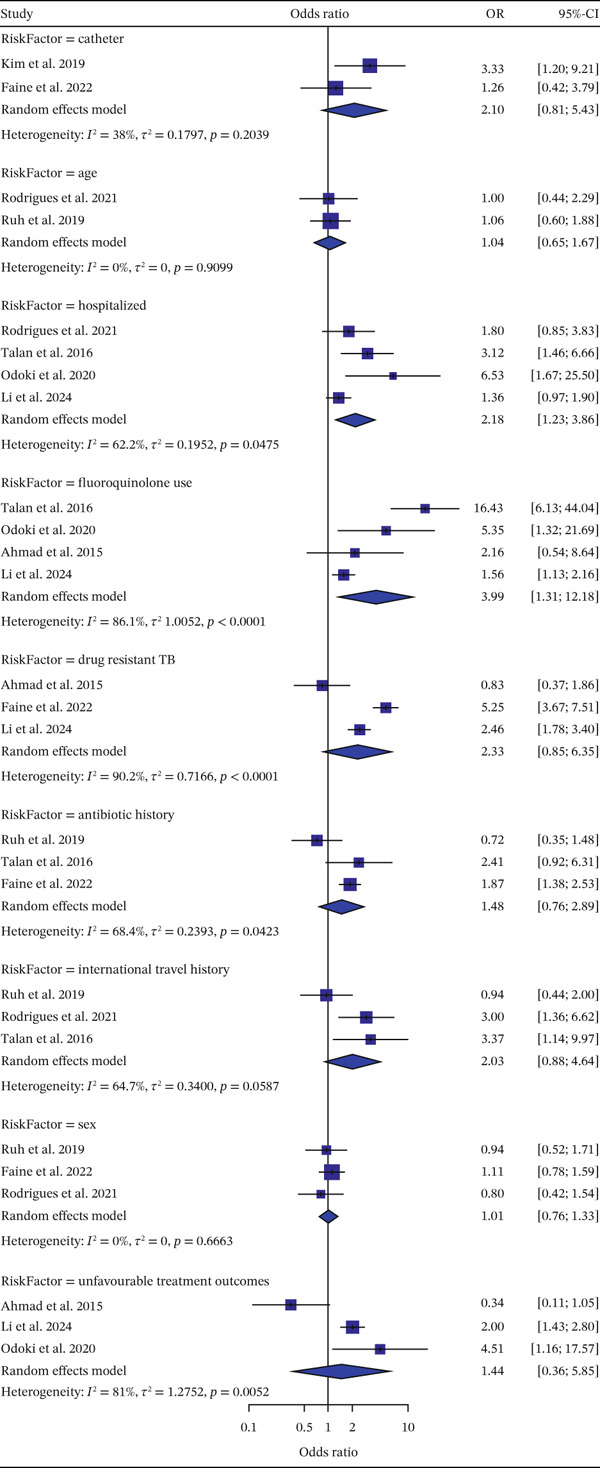
Odds ratio of risk factors associated with FQR. This figure presents study‐level and pooled estimates of the association between selected risk factors and FQR, expressed as odds ratios (ORs) with 95% confidence intervals (CIs). Individual studies are represented by squares (with size proportional to study weight), and horizontal lines indicate 95% CIs. The pooled estimates for each risk factor were calculated using a random‐effects model and are represented by diamonds. The vertical reference line represents an OR of 1.0, indicating no association. Estimates to the right of the line suggest increased odds of fluoroquinolone resistance, while those to the left suggest reduced odds. Between‐study heterogeneity was assessed using the *I* ^2^ statistic and *τ* ^2^.

Finally, three studies investigated the association between international travel history and FQR. However, the pooled analysis showed no significant association (OR = 1.44; 95% CI: 0.36–5.85). The OR of risk factors associated with FQR is shown in Figure 6.

### 3.6. Publication Bias

Visual inspection of the funnel plots for FQR revealed varying degrees of potential publication bias across the seven bacterial species included in the meta‐analysis. The plot for *Campylobacter* spp. showed a clear asymmetry, indicating the possibility of publication bias, particularly favoring studies with higher or lower prevalence estimates. In contrast, the funnel plot for *E. coli* appeared relatively symmetrical, suggesting a lower likelihood of bias, although some minor sparsity on one side could indicate the need for formal statistical confirmation using Egger′s test.

For *Klebsiella* spp., the plot showed moderate asymmetry with fewer studies on the lower left side, hinting at underreporting of studies with lower resistance estimates, potentially influencing the pooled results. The plot for *M. tuberculosis* was highly sparse and asymmetric, strongly suggesting substantial publication bias or high between‐study heterogeneity. Similarly, the plot for studies grouped under “Others” demonstrated an irregular pattern, implying possible bias or the presence of outlier effects due to mixed populations. In contrast, *P. aeruginosa* showed a generally symmetrical funnel shape with minimal signs of small‐study effects, indicating a low likelihood of publication bias. Lastly, the plot for *Proteus* spp. displayed clear asymmetry with a clustering of studies on one side, suggesting publication bias or selective reporting of studies with higher resistance rates.

**Figure 7:**
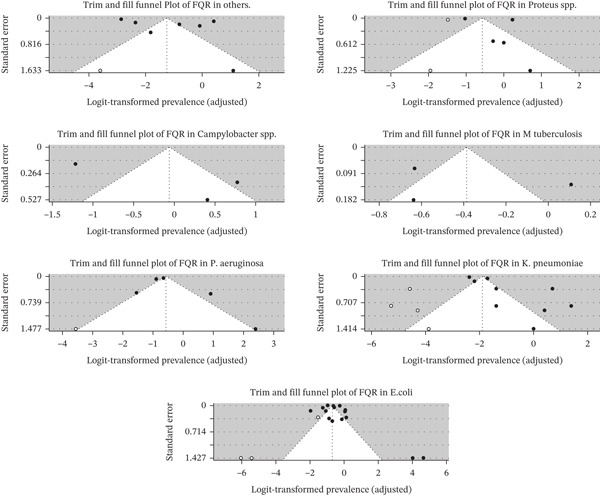
Funnel plots for the publication bias of FQR from each pathogen. Funnel plot of included studies evaluating potential publication bias. The effect size is plotted against standard error for each study. Visual inspection of symmetry indicates the likelihood of bias across studies.

Overall, these findings highlight the importance of complementing visual inspection with statistical methods such as Egger′s test and trim‐and‐fill analysis to assess and correct for potential bias in the pooled prevalence estimates. Funnel plots for the overall FQR and each GNB are shown in Figure 7.

## 4. Discussion

Fluoroquinolones are broad‐spectrum antibiotics widely used for the treatment of both community‐ and hospital‐acquired infections [40] and remain a significant global concern, as reflected in the findings of this systematic review and meta‐analysis. The overall pooled prevalence of FQR among major bacterial pathogens was found to be 35%. This resistance rate is higher than that reported in a previous meta‐analysis conducted in Ethiopia, where the pooled prevalence of resistance among GNBs was approximately 21% [41]. In terms of specific bacterial species, our findings on the pooled prevalence of FQR varied widely, ranging from 23% in *Klebsiella* spp. to 49% in *Campylobacter* spp., with intermediate rates observed in *E. coli*, *M. tuberculosis*, *P. aeruginosa*, *Proteus* spp., and other pathogens. In contrast, a network meta‐analysis reported even higher resistance rates to fluoroquinolones among three GNBs—Enterobacteriaceae (43.1%), *P. aeruginosa* (57.3%), and *A. baumannii* (65.7%)—with the majority of the studies conducted in Europe [42]. Similarly, a meta‐analysis from China reported that ciprofloxacin resistance in *P. aeruginosa* reached approximately 35% [43]. This variation across studies and regions highlights the dynamic and context‐dependent nature of FQR in GNBs and other pathogens, suggesting the need for future research to explore underlying drivers and regional determinants.

However, the pooled analyses demonstrated substantial heterogeneity across studies (*I* ^2^ often exceeding 90%). Such heterogeneity is frequently observed in AMR meta‐analyses due to differences in study design, population characteristics, laboratory methodologies, and regional antimicrobial prescribing practices [6, 44]. On the one hand, the included studies demonstrated substantial methodological variability, particularly in susceptibility testing standards, which may have influenced resistance classification and contributed to heterogeneity in the pooled estimates. Specifically, different studies applied guidelines that define distinct clinical breakpoints for fluoroquinolones. Therefore, the pooled prevalence estimates presented in this study should be interpreted as broad summaries rather than precise global estimates of FQR.

Furthermore, the metaregression analysis indicated that resistance in *E. coli* varied across different fluoroquinolones, with norfloxacin generally showing the highest levels, followed by ciprofloxacin and levofloxacin. Temporal trend analysis was conducted for *E. coli* because it was the most frequently reported pathogen across the included studies, allowing sufficient data points for comparative analysis across time periods. In relation to *E. coli* resistance, our findings indicated a higher prevalence compared to a previous meta‐analysis, which reported resistance rates of 38% in hospital settings and 27% in community‐based settings [45]. This concern is further supported by studies from Vietnam, where resistance reached 69% [46], highlighting regional differences and the potential influence of local prescribing practices and antimicrobial stewardship policies.

Compared to ciprofloxacin, norfloxacin—also a second‐generation fluoroquinolone—demonstrated a higher pooled prevalence of FQR in *E. coli*. This rate is considerably higher than that reported in a previous study, which observed a resistance rate of 47% in *E. coli* isolates from UTIs [47]. In contrast, a study conducted in Ethiopia reported a significantly lower norfloxacin resistance rate in *E. coli*, at around 28% [48]. For the third‐generation fluoroquinolone, levofloxacin, the estimated FQR rate was approximately 39%. However, a study from Vietnam reported a substantially higher resistance rate to levofloxacin in *E. coli*, reaching 64% [46]. These variations in resistance rates across geographic regions underscore the importance of tailoring empiric treatment guidelines to reflect local antimicrobial susceptibility patterns [42]. The growing pace of globalization has also contributed to the international spread of resistant bacterial clones, reinforcing the need for continuous surveillance and updated resistance data both within and across countries over time [42, 49].

Time trend analysis showed slight, nonsignificant increases in resistance for ciprofloxacin and norfloxacin, suggesting no clear evidence of consistent growth, while levofloxacin demonstrated a significant upward trend, indicating a progressive rise in resistance prevalence over time. Overall, these findings suggest the already high levels of FQR in *E. coli*, with levofloxacin resistance showing a potentially increasing trend. However, this observation should be interpreted with caution, given the limited number of studies and the presence of substantial heterogeneity.

Risk factors associated with FQR in major bacterial pathogens include prior fluoroquinolone use and a history of hospitalization. A study from Israel demonstrated that prior use of ciprofloxacin within a 180‐day period was significantly associated with resistance, with an OR of approximately 2.25 [50]. Similarly, a history of hospitalization was found to be a contributing factor to increased FQR, suggesting that hospital‐acquired infections may play a role in driving resistance within the community. One study also noted that antibiotic resistance in the elderly is on the rise due to frequent exposure to healthcare environments, making this population more vulnerable to infections caused by MDR organisms [51]. To address this issue, the implementation of antimicrobial stewardship programs (ASPs) and strict infection control measures is essential. ASPs help to reduce inappropriate antibiotic use, thereby improving treatment outcomes, ensuring patient safety, and reducing healthcare costs [52].

Although age and sex were not statistically significant risk factors in this review, other studies from the United States suggest they may contribute to variability in resistance patterns, particularly in outpatient settings. One such study found that females over the age of 50 had higher odds of ciprofloxacin resistance (OR = 3.04; 95% CI: 2.48–3.74) compared to females under 19 years old. Similarly, males over 50 years had greater odds of resistance (OR = 2.59; 95% CI: 1.18–5.69) than their younger counterparts [53]. These differences may not have been observed in our analysis due to limited sample sizes or variations in the characteristics of included studies.

While several risk factors were identified, the number of studies contributing to certain pooled estimates was limited. Consequently, these findings should be interpreted prudently, given the potential influence of interstudy heterogeneity, regional variability, and methodological constraints, all of which may affect the robustness and generalizability of the results. Further, well‐designed studies are needed to confirm these associations and better understand the underlying drivers of FQR.

## 5. Limitations of the Study

This study has several limitations that should be acknowledged. First, the included studies were conducted using varying methodologies, analytical approaches, and outcome measures, which posed challenges in combining data under consistent subcategories—particularly for specific bacterial species. This heterogeneity limited the ability to perform uniform subgroup analyses and may have affected the precision of pooled estimates.

Moreover, many studies assessed resistance to multiple fluoroquinolones rather than focusing on a single agent, making it difficult to isolate the effect of individual antibiotics within specific subgroups. The other limitation of this study was the inclusion of *M. tuberculosis*, which is taxonomically distinct from GNBs. However, fluoroquinolones play a crucial role in the management of MDR TB, and resistance to these agents represents a major global health concern. Including TB studies allowed the present review to highlight broader patterns of fluoroquinolone resistance across clinically significant bacterial pathogens. Nevertheless, the biological differences between TB and typical Gram‐negative organisms should be considered when interpreting the pooled estimates.

Second, the inclusion criteria were limited to studies published in English, which may have introduced language bias and led to the exclusion of relevant data published in other languages. Additionally, temporal changes in antimicrobial susceptibility testing standards and interpretive criteria across the study periods may have contributed to inconsistencies in the reported resistance rates and influenced the conclusions drawn.

Thirdly, although screening and data extraction were conducted independently by two reviewers, the absence of a third independent adjudicator may represent a methodological limitation and could introduce a small risk of selection bias.

Lastly, several pooled estimates of FQR were derived from studies with relatively small sample sizes and highly variable resistance rates, which could compromise the robustness and generalizability of the findings. These fluctuations highlight the need for cautious interpretation and underscore the importance of conducting larger, standardized, and regionally representative studies in the future.

## 6. Conclusion

Overall, this meta‐analysis revealed a wide range of GNB species with a modest but concerning prevalence of FQR. In the subgroup analysis, norfloxacin showed the highest increase in resistance rates over time. Hospitalization and a history of fluoroquinolone use were identified as key factors associated with the rise in resistance among major pathogens. These findings highlight the urgent need for strengthened ASPs to reduce unnecessary fluoroquinolone use. Enhanced surveillance systems for AMR are also essential to monitor resistance trends and inform evidence‐based prescribing policies. Future research should prioritize standardized surveillance methodologies and longitudinal studies to better understand resistance dynamics across different clinical settings.

## Funding

No funding was received for this manuscript.

## Disclosure

An earlier version of this manuscript was posted as a preprint on Research Square (Anggriawan & Rochmawati, 2025) [54].

## Conflicts of Interest

The authors declare no conflicts of interest.

## Supporting information


**Supporting Information** Additional supporting information can be found online in the Supporting Information section. Table S1: The Newcastle–Ottawa Scale (NOS) to assess the risk of bias of observational studies. Table S2: Characteristics of included studies. Table S3: Characteristics of included studies based on bacterial subgroup.

## Data Availability

The data that support the findings of this study are available from the corresponding author upon reasonable request.
