# Supplementary material for: Prevalence and Risk Factors of Fluoroquinolone Resistance in Major Bacterial Pathogens: A Systematic Review and Meta‐Analysis
Source: Int J Microbiol. 2026 Apr 28;2026:3173058. doi: 10.1155/ijm/3173058 (PMC13122566; doi:10.1155/ijm/3173058)
Supplement: Supplementary file 1 — Supporting Information Additional supporting information can be found online in the Supporting Information section. Table S1: The Newcastle–Ottawa Scale (NOS) to assess the risk of bias of observational studies. Table S2: Characteristics of included studies. Table S3: Characteristics of included studies based on bacterial subgroup. [file IJM-2026-3173058-s001.docx]

**Supplementary Table 1**

**The Newcastle-Ottawa Scale (NOS) to assess the risk of bias of observational studies**

| **Author** | **Item 1** | **Item 2** | **Item 3** | **Item 4** | **Item 5** | **Item 6** | **Item 7** | **Item 8** | **Overall** |
| --- | --- | --- | --- | --- | --- | --- | --- | --- | --- |
| Brintz, et al | (*) | (-) | (*) | (*) | (**) | (*) | (*) | (*) | 8 (*) |
| Martinet, et al | (*) | (-) | (*) | (*) | (*) | (*) | (*) | (*) | 7 (*) |
| Marepalli, et al | (*) | (-) | (*) | (*) | (*) | (*) | (*) | (*) | 7 (*) |
| Odoki et al | (*) | (-) | (*) | (*) | (*) | (*) | (-) | (-) | 5 (*) |
| Ruh et al | (*) | (-) | (*) | (*) | (*) | (*) | (-) | (-) | 5 (*) |
| Zaidi et al | (*) | (-) | (*) | (*) | (*) | (*) | (-) | (-) | 5 (*) |
| Talan et al | (*) | (-) | (*) | (*) | (*) | (*) | (-) | (-) | 5 (*) |
| Zhang et al | (*) | (-) | (*) | (*) | (*) | (*) | (*) | (*) | 7 (*) |
| Li et al | (*) | (-) | (*) | (*) | (-) | (*) | (-) | (*) | 5 (*) |
| Zilberberg et al | (*) | (-) | (*) | (*) | (*) | (*) | (*) | (*) | 7 (*) |
| Banukumar et al | (*) | (-) | (*) | (*) | (-) | (*) | (-) | (*) | 5 (*) |
| Ahmad el al | (*) | (-) | (*) | (*) | (*) | (*) | (-) | (-) | 5 (*) |
| Braam et al | (*) | (-) | (*) | (*) | (*) | (*) | (-) | (-) | 5 (*) |
| Faine et al | (*) | (-) | (*) | (*) | (*) | (*) | (-) | (-) | 5 (*) |
| Deku et al | (*) | (-) | (*) | (*) | (-) | (*) | (-) | (-) | 4 (*) |
| John et al | (*) | (-) | (*) | (*) | (*) | (*) | (*) | (*) | 7 (*) |
| Bidell, et al | (*) | (-) | (*) | (*) | (-) | (*) | (-) | (*) | 5 (*) |
| Baek, et al | (*) | (-) | (*) | (*) | (*) | (*) | (*) | (*) | 7 (*) |
| Chhakchhuak et al | (*) | (-) | (*) | (*) | (-) | (*) | (-) | (*) | 5 (*) |
| Jouhar | (*) | (-) | (*) | (*) | (*) | (*) | (-) | (*) | 6 (*) |
| Poonia | (*) | (-) | (*) | (*) | (-) | (*) | (-) | (*) | 5 (*) |
| Tian | (*) | (-) | (*) | (*) | (-) | (*) | (-) | (-) | 5 (*) |
| Kim | (*) | (-) | (*) | (*) | (*) | (*) | (*) | (*) | 7 (*) |
| Rodrigues et al | (*) | (-) | (*) | (*) | (*) | (*) | (-) | (-) | 5 (*) |

**Supplemantary Table 2**

**Characteristics of included studies**

| **No** | **Authors** | **Year** | **Country** | **Study Design** | **Setting** | **Number of Patients** | **Age (Mean)** | **Patient Diagnose** | **Sample** |
| --- | --- | --- | --- | --- | --- | --- | --- | --- | --- |
| 1 | Brintz, et al (13) | 2024 | USA | Retrospective cohort | Out-patients | 214,656 | 68.9 | UTI | Urine |
| 2 | Martinet, et al (14) | 2024 | France | Retrospective cohort | Hospital | 210 | 63 (51; 69) Median (Q1;Q3) | Acute graft pyelonephritis (AGPN) | NA |
| 3 | Marepalli, et al (15) | 2024 | India | Retrospective observational | Hospital | 280 | 43.5 | UTI | Urine |
| 4 | Li et al (16) | 2024 | China | Retrospective | Hospital | 850 | 39 (28; 55) Median, IQR | TBC | Sputum |
| 5 | Baek, et al (17) | 2024 | South Korea | Retrospective cohort | Hospital | 108 | 59 (37;72) Median (IQR) | Campylobacteriosis | Blood |
| 6 | Chhakchhuak et al (18) | 2023 | India | Retrospective | Hospital | 586 | 40 (20;80) Median (IQR) | UTI | NA |
| 7 | Braam et al (19) | 2022 | Netherland | Cross-Sectional | Sexual Health Centre | 669 | <25 - >45 | Sexual Transmitted infection (STI) | Urine, Swab Anal, Swab Vaginal |
| 8 | Faine et al (20) | 2022 | USA | Observational cohort | Hospital | 3779 | 62,9 (41;77,6) Median, IQR | UTI | urine |
| 9 | Deku et al (21) | 2022 | Ghana | Cross-sectional | Hospital | 135 | 10-60 | UTI | Urine |
| 10 | John et al (22) | 2021 | USA | Retrospective cohort | Hospital | 8680 | 18-80 | Pneumonia | Blood Culture, Respiratory Culture |
| 11 | Rodrigues et al (23) | 2021 | USA | Retrospective | Hospital | 214 | NA | Campylobacteriosis | Stools |
| 12 | Odoki et al (24) | 2020 | Uganda | Cross-sectional | Hospital | 86 | NA | UTI | Urine |
| 13 | Zilberberg et al (25) | 2020 | USA | Cross-sectional | Hospital | 23,331 | NA | UTI | NA |
| 14 | Jouhar et al (26) | 2020 | Lebanon | Retrospective | Medical centre | 356 | 66.9 | Diabetic Foot Ulcer | tissue culture |
| 15 | Ruh et al (27) | 2019 | Cyprus | Cross-sectional | Hospital | 500 | 32.92 | Healthy patient | Rectal swab/ Stool |
| 16 | Kim et al (28) | 2019 | South Korea | Retrospective cohort | Hospital | 130 | 61.22 | BSI | Blood |
| 17 | Poonia et al (29) | 2018 | India | Prospective | Hospital | 100 | 21.4 | Typhoid | Blood |
| 18 | Zaidi et al (30) | 2017 | Pakistan | Retrospective | Hospital | 133 | 16 (10-26) Median (Q1-Q3) | TBC | sputum |
| 19 | Banukumar et al (31) | 2017 | India | Cross-sectional | Hospital | 718 | NA | UTI | Urine |
| 20 | Talan et al (32) | 2016 | USA | cross-sectional | Hospital | 521 | 37 (26 - 52) Median | Pyelonephritis | Urine |
| 21 | Bidell, et al (33) | 2016 | USA | Retrospective observational | Hospital | 9944 | 0-65 | UTI | Urine |
| 22 | Tian et al (34) | 2016 | China | Cross sectional | Hospital | 508 | 0-5 | Acute diarrhea | Stool |
| 23 | Ahmad el al (35) | 2015 | Pakistan | Cross-Sectional | Hospital | 243 | 30.4 | TBC | sputum |
| 24 | Zhang et al (36) | 2015 | China | Retrospective | Hospital | 38 | 55 (Median)  21-75 (Range) | Cancer | Blood |

**Supplemantary Table 3**

**Characteristics of Included studies based on Bacterial subgroup**

| **Authors** | **Year** | **Country** | **Number of Isolates** | **Number of Resistant Prevalent** |
| --- | --- | --- | --- | --- |
| ***Camplylobacter* spp.** |  |  |  |  |
| Tian et al | 2016 | China | 15 | 9 |
| Rodrigues et al | 2021 | USA | 214 | 49 |
| Baek, et al | 2024 | South Korea | 38 | 26 |
| ***Escherichia coli*** |  |  |  |  |
| Zhang et al | 2015 | China | 28 | 13 |
| Bidell, et al | 2016 | USA | 9944 | 3,430 |
| Tian et al | 2016 | China | 24 | 8 |
| Talan et al | 2016 | USA | 397 | 48 |
| Banukumar et al | 2017 | India | 222 | 114 |
| Ruh et al | 2019 | Cyprus | 51 | 51 |
| odoki et al | 2020 | Uganda | 36 | 19 |
| Zilberberg et al | 2020 | USA | 11,510 | 5007 |
| John et al | 2021 | USA | 1029 | 365 |
| Faine et al | 2022 | USA | 1417 | 313 |
| Deku et al | 2022 | Ghana | 135 | 69 |
| Chhakchhuak et al | 2023 | India | 27 | 27 |
| Brintz, et al | 2024 | USA | 213,833 | 58,620 |
| Martinet, et al | 2024 | France | 38 | 11 |
| Marepalli, et al | 2024 | India | 210 | 53 |
| ***Klebsiella* spp.** | | | | |
| Zhang et al | 2015 | China | 2 | 1 |
| Banukumar et al | 2017 | India | 60 | 12 |
| odoki et al | 2020 | Uganda | 10 | 2 |
| Zilberberg et al | 2020 | USA | 3,395 | 518 |
| John et al | 2021 | USA | 696 | 70 |
| Chhakchhuak et al | 2023 | India | 5 | 3 |
| Martinet, et al | 2024 | France | 10 | 8 |
| Marepalli, et al | 2024 | India | 42 | 28 |
| Brintz, et al | 2024 | USA | 83,016 | 7,011 |
| ***Micobacterium tuberculosa*** | | | | |
| Ahmad el al | 2015 | Pakistan | 243 | 128 |
| Zaidi et al | 2017 | Pakistan | 133 | 46 |
| Li et al | 2024 | China | 850 | 295 |
| ***Pseudomonas aeruginosa*** | | | | |
| Banukumar et al | 2017 | India | 21 | 15 |
| Jouhar et al | 2020 | Lebanon | 34 | 6 |
| Zilberberg et al | 2020 | USA | 3,080 | 1058 |
| John et al | 2021 | USA | 1342 | 391 |
| Chhakchhuak et al | 2023 | India | 5 | 5 |
| ***Proteus* spp*.*** | | | | |
| Banukumar et al | 2017 | India | 12 | 6 |
| odoki et al | 2020 | Uganda | 3 | 2 |
| Zilberberg et al | 2020 | USA | 2,739 | 1524 |
| Marepalli, et al | 2024 | India | 14 | 6 |
| Brintz, et al | 2024 | USA | 37,466 | 9,855 |
| **Others** | | | | |
| Tian et al | 2016 | China | 43 | 6 |
| Poonia et al | 2018 | India | 71 | 34 |
| Kim et al | 2019 | South Korea | 126 | 39 |
| Zilberberg et al | 2020 | USA | 473 | 284 |
| Braam et al | 2022 | Netherland | 669 | 58 |
| Chhakchhuak et al | 2023 | India | 1 | 1 |
| Brintz, et al | 2024 | USA | 15,080 | 819 |
